# *ALS* gene overexpression and enhanced metabolism conferring *Digitaria sanguinalis* resistance to nicosulfuron in China

**DOI:** 10.3389/fpls.2023.1290600

**Published:** 2023-11-17

**Authors:** Ruolin Wang, Ying Sun, Yuning Lan, Shouhui Wei, Hongjuan Huang, Xiangju Li, Zhaofeng Huang

**Affiliations:** ^1^ State Key Laboratory for Biology of Plant Diseases and Insect Pests, Institute of Plant Protection, Chinese Academy of Agricultural Sciences, Beijing, China; ^2^ College of Plant Protection, Northeast Agricultural University, Harbin, China

**Keywords:** *Digitaria sanguinalis*, nicosulfuron, herbicide resistance, *ALS* gene overexpression, herbicide metabolism

## Abstract

Crabgrass (*Digitaria sanguinalis*) is a common malignant weed in corn fields in China. Recently, the acetolactate synthase (ALS) inhibitor, nicosulfuron, has shown decreasing efficacy against crabgrass. In order to elucidate the molecular basis of resistance to nicosulfuron in crabgrass, we conducted bioassays, combined with gene sequence analysis, relative expression and relative copy number analysis, to characterize resistance in crabgrass populations collected from Beijing, Heilongjiang, Jilin and Anhui provinces. Whole-plant dose-response results indicated that only population collected in Heilongjiang province (HLJ) had developed low level of resistance to nicosulfuron compared with the sensitive population (SD22). No known resistant mutation of *ALS* gene was found in HLJ population. The real-time fluorescence quantitative PCR results showed that the *ALS* gene copy number did not differ significantly between the HLJ and SD22 populations. However, the *ALS* gene expression in the HLJ was 2.07-fold higher than that of the SD22 population at 24 h after treatment with nicosulfuron. Pretreatment with the cytochrome P450 (CYP450) inhibitor malathion, piperonyl butoxide (PBO), and the glutathione S-transferase (GST) inhibitor 4-Chloro-7-nitro-1,2,3-benzoxadiazole (NBD-Cl) all partially reversed HLJ resistance. Among them, the synergistic effect of PBO and nicosulfuron is the most significant. This is the first report of resistance to nicosulfuron in crabgrass through *ALS* gene overexpression and possible metabolic resistance.

## 1 Introduction

Acetolactate synthase (ALS), also known as acetohydroxyacid synthase (AHAS), plays a crucial role in the biosynthetic pathway of the branched-chain amino acids valine, leucine, and isoleucine (Umbarger, 1978). Additionally, it serves as the target for five categories of ALS-inhibiting herbicides, namely sulfonylurea (SU), triazolopyrimidine (TP), imidazolinone (IMI), sulfonylaminocarbonyltriazolinone (SCT) and pyrimidinylthiobenzoate (PTB). ALS-inhibiting herbicides impede the biosynthesis of branched-chain amino acids and their associated proteins, which can affect plant growth and lead to plant death. Since first commercialization in 1982, ALS inhibitors have been widely utilized in weed control of wheat, corn and other crop fields due to their broad spectrum, low toxicity and broad crop selectivity (Mazur and Falco, 1989; Tranel and Wright, 2002). However, less than a year after the ALS inhibitor was applied, there was report of *Lolium rigidum* developing resistance to it (Heap, 2023). And by 1998, ALS inhibitor-resistant weed species outnumbered all other herbicides (Tranel and Wright, 2002). As of today, there are 172 ALS inhibitor-resistant weed species worldwide, including 105 dicots and 67 monocots weeds (Heap, 2023).

Weed resistance to ALS inhibitors is mainly caused by target-site resistance (TSR) induced by single amino acid substitution. At present, nine amino acid substitution sites have already been identified in ALS inhibitor-resistant weeds: Ala-122, Pro-197, Ala-205, Phe-206, Asp-376, Arg-377, Trp-574, Ser-653 and Gly-654 (Fang et al., 2022; Heap, 2023). Among resistant weeds, the most common was the Pro-197 mutation, followed by mutation at Trp-574 (Heap, 2023). Moreover, both amino acid substitution at different sites of ALS and different amino acid substitutions at the same site lead to different degrees of weed resistance (Powles and Yu, 2010; Yu and Powles, 2014). More problematically, mutations in the target gene may make the weed cross-resistant to different herbicides with the same mode of action. Five different amino acid substitutions at the Pro-197 site in *ALS* gene confer cross-resistance to SU and IMI herbicides in *Lactuca serriola* (Merriam et al., 2023). Similarly, cross-resistance of *Amaranthus palmeri* to the ALS inhibitors thifensulfuron-methyl and imazamox is due to its Trp-574-Leu substitution (Manicardi et al., 2023). In addition, overexpression of *ALS* gene, a less common mechanism of TSR, may also induce resistance to ALS-inhibiting herbicides in weeds (Sen et al., 2021).

Although most cases of resistance were caused by TSR, non-target-site resistance (NTSR) is also an important mechanism for weed resistance to ALS-inhibiting herbicides. The NTSR mechanism is usually to enhance the metabolism and sequestration of herbicides, and reduce the absorption and transport of herbicides. Of these, metabolism-based NTSR is the most common and is associated with increased activity of enzyme complexes such as cytochrome P450 monooxygenases (CYP450s), glutathione S-transferases (GSTs), glucosyltransferase (GTs), ATP-binding cassette (ABC) transporters (Iwakami et al., 2014; Zhao et al., 2017). Pretreatment with malathion (a CYP450 inhibitor) could reverse weed resistance to sulfosulfuron and penoxsulam, which suggests that CYP450s are involved in metabolic resistance (Owen et al., 2012; Riar et al., 2012). NTSR can confer weed resistance to a variety of herbicides with the same or different sites of action, sometimes even non-commercial herbicides (Ma et al., 2013; Concepcion et al., 2021). For example, the *CYP81A10v7* gene confers cross-pollinated *Lolium rigidum* multiple-resistance to herbicides with five modes of action: ALS, photosystem II (PS II), 4-hydroxyphenylpyruvate dioxygenase (HPPD), acetyl-CoA carboxylase (ACCase) and tubulin inhibitors (Han et al., 2021). The innate resistance of *Centaurea* to tribenuron-methyl and the cross-resistance to the SU, TP and IMI herbicides are mainly due to metabolism of herbicides by CYP450 and reduced absorption and transport (Palma-Bautista et al., 2023).

Crabgrass (*Digitaria sanguinalis*) is a malignant weed in autumn ripe dry field, mainly harming corn, soybean, cotton, sorghum and other crops. Herbicides have been the main strategy for controlling crabgrass over that last decades. However, the heavy use of herbicide has resulted in the selection and evolution of resistant crabgrass populations (Powles and Yu, 2010). At present, crabgrass has been documented to develop resistance to four categories of herbicides: ALS, PS II, ACCase and 5-enolpyruvylshikimate-3-phosphate synthase (EPSPS) inhibitors (Heap, 2023). Nicosulfuron, an ALS inhibitor, has played an important role in the control of crabgrass since it was registered and popularized in China in 1990s. Unfortunately, recent studies on the resistance of crabgrass to nicosulfuron have been frequently reported, and the mechanisms of resistance include target gene mutations and enhanced metabolism mediated by CYP450s or GSTs (Zhang et al., 2013; Li et al., 2017; Mei et al., 2017; Zhao et al., 2023).

Monitoring the distribution of resistant weeds in the field and revealing their potential resistance mechanisms are of great importance for prolonging the use life of herbicides and formulating weed control strategies. Accordingly, the purpose of this study was to (1) determine the resistance level of crabgrass populations to nicosulfuron in different regions of China; (2) identify the resistance mechanism of crabgrass to nicosulfuron; and (3) investigate the sensitivity of crabgrass to other herbicides.

## 2 Materials and methods

### 2.1 Plant material

The seeds of four suspected nicosulfuron-resistant crabgrass populations were collected from Beijing, Heilongjiang, Jilin and Anhui provinces in China, where the crabgrass could not be controlled by nicosulfuron at recommended field doses (60 g ai ha^-1^). The seeds of SD22 population provided by Weed Science Department, Institute of Plant Protection, Shandong Academy of Agricultural Sciences were used as control (**Table 1**). Seeds from all populations were soaked with gibberellin (400 mg L^-1^) for 24 h to break dormancy. Twenty seeds were seeded in 9 cm diameter plastic pots containing moist soil substrate, and thinned out to eight plants per pot after emergence. Plants were grown in a greenhouse at 30/20°C day/night temperature and 16/8 h photoperiod, and regularly watered and fertilized from the bottom.

**Table 1:** Information of *Digitaria sanguinalis* populations in whole-plant dose-response experiment.

Population	Location	Co-ordinate (E, N)	Herbicide applied history
HLJ	Kaiyuan, Heilongjiang	124°2’57”, 42°36’33”	Over 5 years of nicosulfuron and mesotrione
BJ	Beijing	116°4’14”, 39°44’29”	Over 5 years of nicosulfuron and mesotrione
AH	Fuyang, Anhui	115°49’13”, 32°53’45”	Over 5 years of nicosulfuron and mesotrione
JL	Songyuan, Jilin	124°50’19.68”, 45°6’37”	Over 5 years of nicosulfuron and mesotrione
SD22	Jinan, Shandong	116°49’11.99”, 36°30’36”	Without any herbicides

### 2.2 Whole-plant dose-response assay

To identify the level of resistance in crabgrass, seedlings were treated with nicosulfuron at 3-4 leaf stage in a spray chamber provided with 450 L ha^-1^ spray solution (3WP-2000, Nanjing Institute of Agricultural Mechanization, Ministry of Agriculture and Rural Affairs, China). Based on preliminary screening tests (**Supplementary Table 1**), nicosulfuron was applied to five populations at doses of 0, 15, 30, 60, 120, 240, 480 g ai ha^-1^ (The recommended field dose is 60 g ai ha^-1^). At 21 days after treatment (DAT), the aboveground tissues of the plants were collected and measured for fresh weight. Seedlings were considered alive if they could produce healthy young leaves. The experiment was carried out twice, and each dose of herbicide was repeated three times with eight plants in each replicate.

### 2.3 *ALS* gene isolation and sequencing

Fresh leaf tissue of 10 surviving plants treated with herbicide were collected from each population. The extraction of genomic DNA was performed using DNAsecure Plant Kit (TianGen Biotech, Co.,Ltd, Beijing, China). Two pairs of primers selected from the previous study (Li et al., 2017; Mei et al., 2017) and a pair of primers designed by Oligo 7 software according to the *ALS* gene sequence of crabgrass (GenBank: KX461957.1) were used for amplifying conserved regions of the *ALS* gene sequence, including nine known resistance mutation sites (**Table 2**). The 20 µL polymerase chain reaction (PCR) system consisted of 0.5 µL each of forward and reverse primers, 10 µL of 2× Tag PCR MasterMix II (TianGen Biotech, Co.,Ltd, Beijing, China), 8 µL of ddH_2_O and 1 µL of genomic DNA. The PCR reaction procedure was as follows: 3 min at 94°C; then 30 cycles: 30s at 94°C, 30 s at 53°C, 1 min at 72°C; and finally extend at 72°C for 5 min. PCR products were sent to Biomed (Beijing, China) for sequencing.

**Table 2:** Primer information used in this study.

Primer	Sequence (5'-3')	Tm (°C)	Purpose
MT-F16^a^	CGACGTCTTCGCCTACCC	57.4	Amplifying *ALS* gene fragments covering Ala-122, Pro-197, Ala-205, and Phe-206
MT-R16^a^	AGCCATCTGCTGTTGGATGT	53.8	
ALS-F5	CACAACTACCTGGTCCTCG	55.5	Amplifying *ALS* gene fragments covering Asp-376, Arg-377
ALS-R5	AGAAGATCATTCATGCCCT	49.0	
MT-B1^b^	GTGGATAAGGCCGACCTGTTGCT	62.3	Amplifying *ALS* gene fragments covering Trp-574, Ser-653, Gly-654
MT-B2^b^	CTGCCATCACC(A/G)TCCAGGATCAT	60.7	
ALS-qF1	GTGTATGCAAATTATGCGGTGGA	59.9	qPCR
ALS-qR1	ACCAATCTCAGCTGGATCAATGT	60.0	
UBQ-qF1	CTCACCGGGAAGACTATCACC	59.6	qPCR normalisation
UBQ-qR1	ATGCCCTCCTTGTCTTGAATCTT	60.0	
Actin-qF1	GTTATCACAATCGGTGCAGAGAG	59.5	qPCR normalisation
Actin-qR1	AACATAGTAGAGCCACCACTGAG	59.5	
18S rRNA-qF3	TCTGCTCGCTGATCCGATGAT	61.4	qPCR normalisation
18S rRNA-qR3	TCAGGCTCCCTCTCCGGAAT	62.2	
GAPDH-qF1	GCGTCAACGAGAAGGAATACAAG	59.9	qPCR normalisation
GAPDH-qR1	CCCATCAACAGTCTTCTGGGTAG	60.4	

a Source from Mei et al., 2017.

b Source from Li et al., 2017.

### 2.4 *ALS* gene expression

At 3-4 leaf stage, HLJ and SD22 populations were sprayed with the recommended field dose of nicosulfuron, and water was sprayed as the control. Samples were taken from the two newly grown leaves at 24 h after treatment. Each treatment consisted of four biological replicates and three technical replicates. In order to regulate the expression of *ALS*, the reference gene was selected from *β-Actin*, *18S rRNA*, ubiquitin (*UBQ*) and glyceraldehyde-3-phosphate dehydrogenase (*GAPDH*) using BestKeeper software. The primers used in the experiment were all designed by Oligo 7 software (**Table 2**), and the amplification efficiency was verified by experiments to be 90%-110%, R^2^>0.99. Total RNA was extracted from the sample using FastPure Universal Plant Total RNA Isolation Kit (Vazyme Biotech Co.,Ltd, Nanjing, China). RNA integrity was assessed using 1% agarose gel electrophoresis, and RNA concentration and purity were measured using nanodrop (NanoDrop, UV). Subsequently, cDNA was synthesized from 1000 ng total RNA using TransScript^®^ All-in-One First-Strand cDNA Synthesis SuperMix for qPCR (One-Step gDNA Removal) (TransGen Biotech Co.,Ltd, Beijing, China). Thirty-two crabgrass samples were analyzed by Quantitative Real-time PCR (qPCR) using ABI 7500 (Applied Biosystems, USA). The qPCR reaction was performed in a 20 μL system containing 10 μL 2× TransStart^®^Top Green qPCR SuperMix (TransGen Biotech Co.,Ltd, China), 0.4 μL each of forward and reverse primer, 8.2 μL Nuclease-Free-water and 1 μL cDNA. The reaction procedure consists of 94°C for 30 s; then 30 cycles of: 94°C for 5 s, 60°C for 34 s. The relative expression of *ALS* gene in crabgrass was calculated by 2^-ΔΔCT^ (Livak and Schmittgen, 2001).

### 2.5 *ALS* gene relative copy number

The genomic DNA extracted from HLJ and SD22 in the previous experiment were analyzed by qPCR with the above primers to detect the copy number of *ALS* gene relative to *UBQ* gene. The reaction procedure and system are the same as the above experiment, and the 20 μL reaction system contains 50 ng genomic DNA. The experiment was conducted twice with four biological replicates each time.

### 2.6 Effects of cytochrome P450 and glutathione S-transferase inhibitors on crabgrass resistance to nicosulfuron

In order to detect the role of CYP450s and GSTs on the resistance of crabgrass to nicosulfuron, the whole-plant dose-response experiment was performed after pretreatment with malathion, piperonyl butoxide (PBO) and 4-Chloro-7-nitro-1,2,3-benzoxadiazole (NBD-Cl). Malathion (1000 g ai ha^-1^), PBO (2100 g ai ha^-1^ for two consecutive times) and NBD-Cl (270 g ai ha^-1^) were sprayed 1 h, 1 h and 48 h before nicosulfuron application, respectively. Both PBO and NBD-Cl were emulsified with 5% dimethyl sulfoxide and 0.1% Tween 80. The doses of nicosulfuron were identical to the whole-plant dose-response experiment described above. Plants treated with a mixture of 5% dimethyl sulfoxide, 0.1% Tween 80 and water were used as controls. The fresh weight of aboveground tissue were measured at 21 DAT. The experiment was conducted twice, and each dose of herbicide was repeated three times with eight plants in each replicate.

### 2.7 Sensitivity of crabgrass to other herbicides.

The sensitivity of HLJ and SD22 populations was determined by applying five herbicides with different modes of action (**Table 3**). Among them, post-emergence treatment herbicides were sprayed at the growth stage of 3-4 leaves, and pre-emergence treatment herbicides were applied when weeds were just sprouting. All herbicides were sprayed using the spray chamber mentioned above. At 21 DAT, the resistance level of crabgrass was evaluated according to plant survival rate. At the recommended field dose, survival rates greater than 80% are considered highly resistant, 30-80% are considered low resistant, and less than 30% are considered sensitive (Huang et al., 2019).

**Table 3:** Herbicide information for cross-resistance and multiple-resistance experiments.

Target^a^	Herbicide	Dose^b^ (g a.i. ha^−1^)	Application method
ALS	Rimsulfuron	11.25, **22.50**, 45.00	Post-emergence treatment
EPSPS	Glyphosate	337.50, **675.00**, 1350.00	Post-emergence treatment
HPPD	Topramezone	13.50, **27.00**, 54.00	Post-emergence treatment
	Mesotrione	56.25, **112.50**, 225.00	Post-emergence treatment
VLCFA	Acetochlor	562.50, **1125.00**, 2250.00	Pre-emergence treatment

a ALS, acetolactate synthase; EPSPS, 5-enolpyruvylshikimate-3-phosphate synthase; HPPD, 4-hydroxyphenylpyruvate dioxygenase; VLCFA, very Long Chain Fatty Acid Synthesis.

b Bold numbers indicate the recommended field dose.

### 2.8 Statistical analysis

Dose-response data from two replicate experiments were analyzed using SigmaPlot 14 software. Nonlinear regression analysis of the four-parameter log-logistic model was used to calculate the dose of herbicide that reduced above-ground portion growth by 50% (GR_50_):


$$y=C+\left[\frac{D-C}{1+{\left(x/GR_{50}\right)}^{b}}\right]$$

In the above formula, *x* is the applied dose of herbicide; *C* is the lower limit of reaction; *D* is the upper limit of the reaction; *b* is the slope of the curve and *y* is the fresh weight of the corresponding dose. The resistance index (RI) was calculated based on the GR_50_ of resistant and susceptible populations:


$$\text{RI}=\frac{GR_{50}\left(R\right)}{GR_{50}\left(S\right)}$$

## 3 Results

### 3.1 Dose Response of crabgrass to nicosulfuron

**Figure 1:**
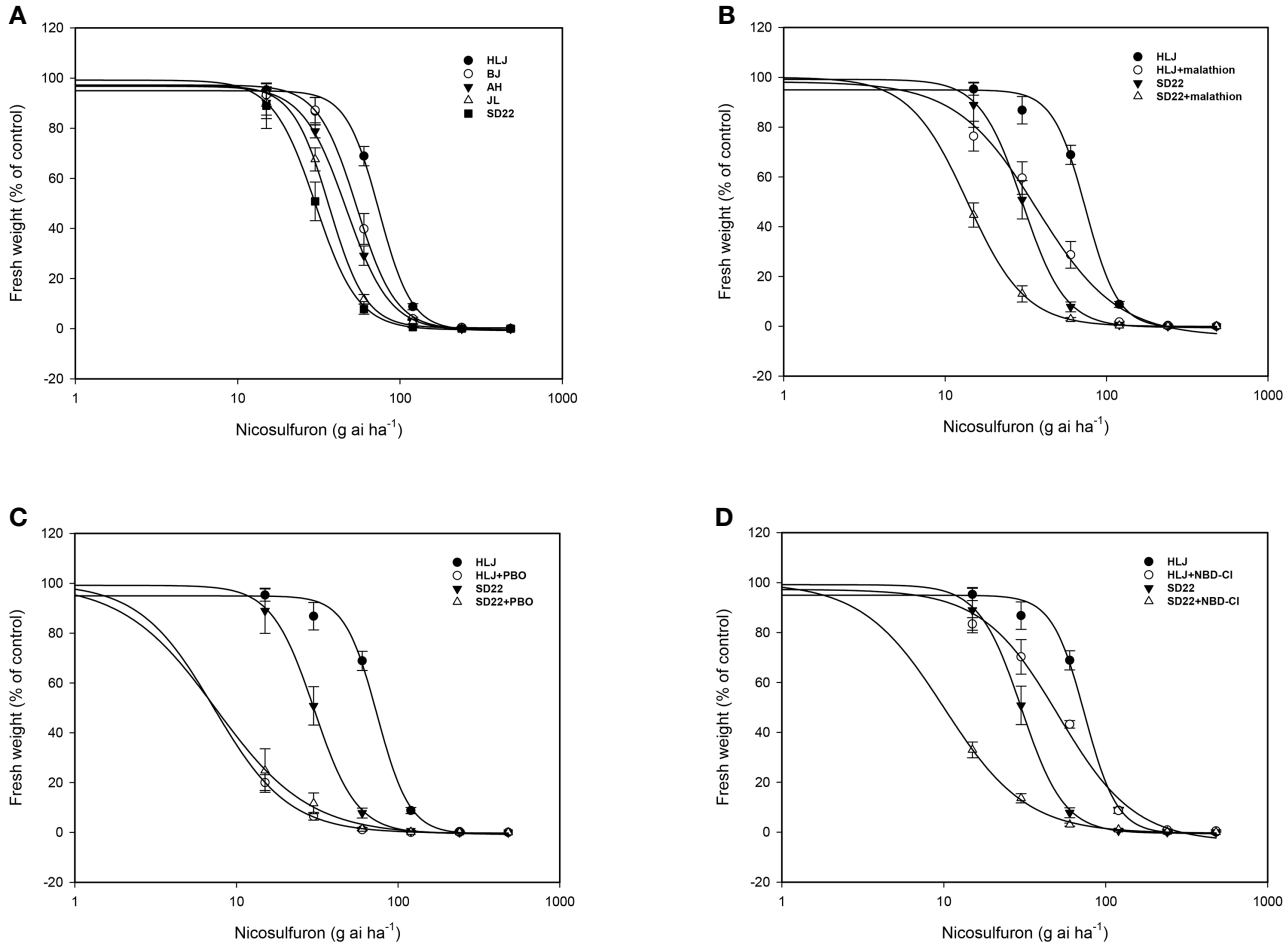
Fitted logarithmic dose-response curves for *Digitaria sanguinalis* populations. **(A)** nicosulfuron, **(B)** nicosulfuron with or without cytochrome P450 (CYP450) inhibitor malathion, **(C)** nicosulfuron with or without CYP450 inhibitor piperonyl butoxide (PBO), **(D)** nicosulfuron with or without glutathione S-transferase inhibitor 4-Chloro-7-nitro-1,2,3-benzoxadiazole (NBD-Cl). The error bar stands for standard error of the mean.

At 21 DAT with the recommended field dose of nicosulfuron, the SD22 population was almost completely controlled, with a 92.22% reduction in fresh weight, and the fresh weight of JL, AH and BJ populations were also reduced by more than 60%; however, the HLJ population was not seriously damaged and the fresh weight decreased by only 31.12%. The dose-response results showed that the HLJ population developed low level resistance to nicosulfuron with the RI of 2.45 (**Table 4**; **Figure 1A**).

**Table 4:** Parameter values of the four-parameter log-logistic equation for calculating GR_50_ values of *Digitaria sanguinalis* populations in whole-plant dose–response experiment.

Population	Regression Parameters^a^				GR_50_ ^b^ ± SE^c^ (g a.i. ha^−1^)	RI^d^
	c	d	b	R^2^		
HLJ	-0.60 ± 3.59	94.95 ± 2.93	-4.32 ± 0.93	0.9973	74.20 ± 4.88	2.45
BJ	-0.34 ± 1.64	97.22 ± 1.83	-3.63 ± 0.43	0.9993	54.23 ± 1.76	1.79
AH	-0.74 ± 2.11	96.75 ± 2.55	-3.22 ± 0.40	0.9988	46.61 ± 2.26	1.54
JL	0.32 ± 1.87	97.04 ± 1.53	-3.84 ± 0.50	0.9989	36.67 ± 1.54	1.21
SD22	-0.43 ± 0.74	99.18 ± 1.14	-3.30 ± 0.19	0.9998	30.23 ± 0.52	–

a $y=c+\left(d-c\right)/\left[1+{\left(x/GR_{50}\right)}^{b}\right]$ , where x is the applied dose of herbicide, c is the lower limit of reaction; d is the upper limit of the reaction; b is the slope of the curve and y is the fresh weight of the corresponding dose.

b GR_50_, herbicide dose that reduces fresh weight by 50%.

c SE, standard error of the mean.

d RI, the resistance index.

The symbol "-" represents a null value.

### 3.2 *ALS* gene sequence analysis

To identify whether *ALS* gene mutations involved in the resistance of crabgrass to nicosulfuron, three pairs of primers were utilized for amplifying the *ALS* gene fragments with sizes of 395 bp, 1125 bp and 876 bp, respectively. Subsequently, three fragments from the same genomic DNA were assembled into a 1647 bp sequence and its amino acid sequence was predicted using Mega 7 software. In previous study, Trp-574 amino acid substitution was reported in crabgrass (Li et al., 2017), but no mutation at this site was detected in the HLJ population in this study. Moreover, no amino acid substitution in the HLJ population was identified at eight other sites which confer resistance to ALS inhibitors in other weeds (GeneBank: OR640488; **Supplementary Figure 1**). This result was confirmed in all tested crabgrass plants.

### 3.3 Relative expression level and copy number of *ALS* gene

**Figure 2:**
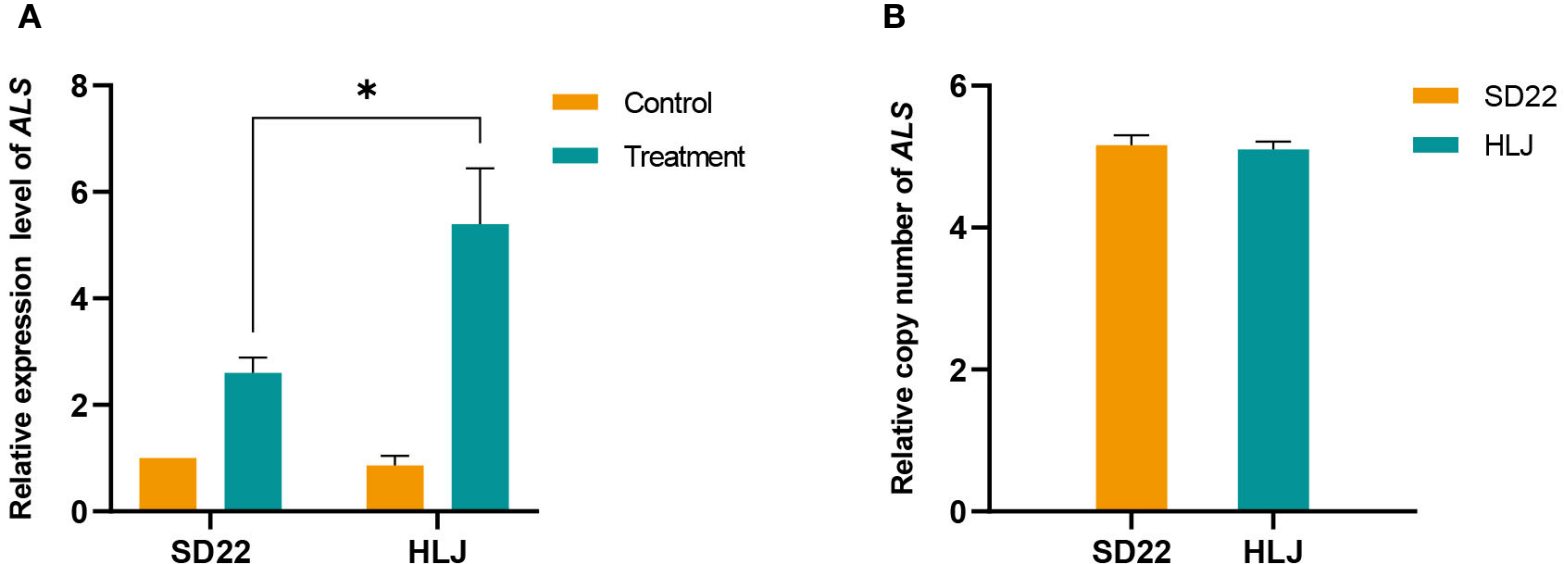
Relative expression and copy number of acetolactate synthase gene (*ALS*) in *Digitaria sanguinalis*. **(A)** Relative *ALS* gene expression level. **(B)** Relative *ALS* gene copy number. The statistical method used for expression analysis was Tukey test, and each treatment included four biological replicates. The statistical method used for copy number analysis was Student’s t test. Each treatment included eight biological replicates. *stands for significant at the 5% level. The error bar stands for standard error of the mean.

*UBQ* was identified as the best reference gene for normalizing *ALS* gene expression (**Supplementary Table 2**). The *ALS* gene expression did not differ significantly between HLJ and SD22 populations when nicosulfuron was not applied. However, the HLJ population exhibited a significant increase in *ALS* gene expression at 24 h after treatment with nicosulfuron(P=0.0003<0.001), which was 2.07-fold higher compared to the SD22 population (P=0.0119<0.05, **Figure 2A**). Moreover, the *ALS* gene copy number of HLJ population was basically the same as that of SD22 population (**Figure 2B**), indicating that the increase of *ALS* expression in HLJ population was induced by herbicide rather than by copy number variation.

### 3.4 Effects of cytochrome P450 and glutathione S-transferase inhibitors pretreatment on nicosulfuron resistance in crabgrass

There was no significant difference in biomass between malathion, PBO and NBD-Cl treated alone and untreated control (P_malathion_=0.453, P_PBO_=0.937, and P_NBD-Cl_ =0.908>0.05). However, both CYP450 inhibitor malathion and PBO pretreatment can significantly increase the toxicity of nicosulfuron to HLJ and SD22 populations (**Table 5**; **Figures 1B, C**). The synergistic effect of PBO and nicosulfuron was more significant, which reduced the fresh weight of HLJ and SD22 populations by 90.22% and 75.29%, respectively. Similarly, GST inhibitor significantly increased the sensitivity of both populations to nicosulfuron, reducing their fresh weight by 31.02% and 66.79%, respectively (**Table 5**; **Figure 1D**). These results indicate that CYP450s and GSTs may be involved in the metabolism of crabgrass to nicosulfuron.

**Table 5:** GR_50_ value of HLJ and SD22 populations treated with nicosulfuron in the presence or absence of cytochrome P450 (CYP450) and glutathione S-transferase (GST) inhibitors.

Treatments	Type of inhibitor	HLJ		SD22		RI
		GR_50_ ± SE	GR_50_ Reduction(%)	GR_50_ ± SE	GR_50_ Reduction(%)	
Nicosulfuron	–	74.20 ± 4.88	–	30.23 ± 0.52	–	2.45
Nicosulfuron + Malathion	CYP450	37.10 ± 4.60	50.00	13.74 ± 0.04	54.55	2.70
Nicosulfuron+PBO	CYP450	7.26 ± 0.31	90.22	7.47 ± 0.96	75.29	0.97
Nicosulfuron+NBD-Cl	GST	51.18 ± 6.02	31.02	10.04 ± 0.34	66.79	5.09

The symbol "-" represents a null value.

### 3.5 Sensitivity of crabgrass to other herbicides.

The experimental results of sensitivity of HLJ and SD22 populations to other herbicides are shown in **Table 6**. HLJ and SD22 could not be completely controlled at the recommended field dose of rimsulfuron, topramezone and mesotrione. However, both populations remained sensitive to glyphosate and acetochlor, with near-zero survival at the recommended field dose of the herbicide.

**Table 6:** Plant survival rate of *Digitaria sanguinalis* population at 21 days after treatment.

Herbicide	Dose^a^ (g a.i. ha^−1^)	survival rate (%) ± SE	
		HLJ	SD22
Rimsulfuron	11.25	100.00 ± 0.00	100.00 ± 0.00
	**22.50**	88.89 ± 3.93	66.67 ± 6.80
	45.00	0.00 ± 0.00	0.00 ± 0.00
Topramezone	13.50	79.17 ± 4.17	100.00 ± 0.00
	**27.00**	37.50 ± 0.00	70.83 ± 8.33
	54.00	0.00 ± 0.00	0.00 ± 0.00
Mesotrione	56.25	100.00 ± 0.00	75.00 ± 7.22
	**112.50**	56.25 ± 15.31	43.75 ± 5.10
	225.00	0.00 ± 0.00	0.00 ± 0.00
Acetochlor	562.50	20.83 ± 2.95	0.00 ± 0.00
	**1125.00**	8.33 ± 5.89	0.00 ± 0.00
	2250.00	0.00 ± 0.00	0.00 ± 0.00
Glyphosate	337.50	0.00 ± 0.00	0.00 ± 0.00
	**675.00**	0.00 ± 0.00	0.00 ± 0.00
	1350.00	0.00 ± 0.00	0.00 ± 0.00

a Bold numbers indicate the recommended field dose.

## 4 Discussion

Herbicide resistance in crabgrass is a growing problem worldwide. Gadamski et al. (1996) found atrazine-resistant crabgrass in Polish orchards; Hidayat and Preston (1997) identified crabgrass showing multiple resistance to ACCase and ALS inhibitors in onion fields in South Australia; Subsequently, Volenberg and Stoltenberg (2002) and Laforest et al. (2017) reported that crabgrass evolved cross-resistance to ACCase inhibitors in corn fields in the United States and onion-carrot rotation fields in Canada, respectively; More recently, Yanniccari et al. (2022) reported that crabgrass in soybean fields in Argentina developed resistance to glyphosate. Nicosulfuron has been utilied for weed control in corn fields for more than 30 years since its registration in China. Regrettably, in recent years, there have been several reports about the decline of nicosulfuron control effect and the evolution of resistance of crabgrass to nicosulfuron in corn fields in Shandong, Shaanxi, Northeast and North China (Zhang et al., 2013; Mei et al., 2017). To better define the distribution and potential resistance mechanism of nicosulfuron-resistant crabgrass populations in China, we characterized the resistance of crabgrass populations from Beijing, Heilongjiang, Jilin and Anhui provinces. Whole-plant dose-response tests showed that only HLJ population had evolved low levels of resistance to nicosulfuron (**Table 4**; **Figure 1A**).

It has been reported that the resistance of most weeds to ALS-inhibiting herbicides is caused by mutations in target gene *ALS*. In crabgrass, the Trp-574-Arg and Asp-376-Glu substitutions of ALS caused high and moderate levels of resistance to ALS-inhibiting herbicides, respectively (Li et al., 2017; Zhao et al., 2023). However, in this study, the HLJ population, which has shown low level of resistance to nicosulfuron, had not known resistance mutations detected in the *ALS* gene. So far, gene duplication was previously seen only in glyphosate-resistant weeds. Glyphosate-resistance caused by *EPSPS* gene duplication was demonstrated in a variety of weeds such as *Amaranthus palmeri* (Gaines et al., 2010), *Amaranthus tuberculatus* (Lorentz et al., 2014), *Amaranthus spinosus* (Nandula et al., 2014), *Kochia scoparia* (Wiersma et al., 2015), *Poa annua L.* (Brunharo et al., 2019), *Chloris truncata* (Ngo et al., 2018), *Bromus diandrus* (Malone et al., 2016), *Lolium perenne ssp. Multiflorum* (Salas et al., 2012), *Salsola tragus* (Yanniccari et al., 2023) and *Eleusine indica* (Chen et al., 2015). However, Laforest et al. (2017) reported *ACCase* gene amplification conferred resistance to ACCase-inhibiting herbicide on crabgrass, indicating that weeds have evolved mechanisms to increase the target gene copy number as resistance to other types of herbicides. Target gene expression and copy number were positively correlated in many weed species (Gaines et al., 2010; Lorentz et al., 2014; Nandula et al., 2014; Chen et al., 2015; Wiersma et al., 2015; Dillon et al., 2017). In our study, the *ALS* gene expression in HLJ was significantly higher than that in SD22 after nicosulfuron treatment, and the relative copy number precludes the possibility of gene amplification (**Figure 2**), suggesting that the induced increase of *ALS* gene expression may be the cause of HLJ resistance to nicosulfuron. Similarly, ALS inhibitor-resistant *Bromus sterilis* was found to have enhanced *ALS* expression in the absence of gene duplication (Sen et al., 2021).

The CYP450 inhibitors malathion, PBO, and the GST inhibitor NBD-Cl are commonly used to detect the metabolic resistance of weeds to herbicides. Mei et al. (2017) and Hidayat and Preston (2001) demonstrated that malathion reversed nicosulfuron resistance in crabgrass; Zhao et al. (2023) found that the combination of NBD-Cl and nicosulfuron increased crabgrass susceptibility; Yang et al. (2023) also reported that PBO pretreatment significantly increased the sensitivity of *Digitaria ciliaris* var. *chrysoblephara* to haloxyfop-P-methyl. Previous studies have shown that compared with the sensitive population, the GSTs activity of methyl fluorophosphate-resistant crabgrass population is higher (Zong et al., 2022), and it was subsequently proved that the increase in GSTs activity was caused by the high expression of the *DsGSTU1* (a tau class plant cytosolic GST) gene (Liu et al., 2023). In this study, malathion, PBO and NBD-Cl pretreatment all increased the sensitivity of crabgrass to nicosulfuron (**Table 5**; **Figures 1B–D**), indicating the presence of NTSR mediated by CYP450s and GSTs in HLJ population. Compared with malathion and NBD-Cl, PBO showed a more significant reversal of crabgrass resistance, indicating that CYP450s inhibited by PBO may play a greater role in the metabolism of crabgrass to nicosulfuron. In addition, since malathion and NBD-Cl pretreatment increased the sensitivity of HLJ and SD22 populations in similar proportions, suggesting that this may be a basal metabolism independent of resistance mechanisms. Therefore, we need to conduct metabolic experiments in the future to prove the existence of metabolic resistance and verify the specific enzymes and metabolites related to the metabolism of nicosulfuron.

In conclusion, this study reports for the first time that crabgrass develops resistance to ALS-inhibiting herbicide through target gene overexpression and possible enhanced metabolism. This study not only enriched the resistance mechanism of crabgrass to nicosulfuron, but also provided a theoretical basis for the integrated control of resistant weeds. However, the regulatory mechanism of *ALS* gene overexpression, specific genes involved in nicosulfuron metabolism and their metabolites still need to be further investigated.

## Data availability statement

The original contributions presented in the study are publicly available. This data can be found here: https://www.ncbi.nlm.nih.gov/nuccore/OR640488.1/.

## Author contributions

RW: Data curation, Investigation, Writing – original draft. YS: Writing – review & editing. YL: Writing – review & editing. SW: Writing – review & editing. HH: Writing – review & editing. XL: Writing – review & editing. ZH: Conceptualization, Methodology, Writing – review & editing.
