# Supplementary material for: ALS gene overexpression and enhanced metabolism conferring Digitaria sanguinalis resistance to nicosulfuron in China
Source: Front Plant Sci. 2023 Nov 17;14:1290600. doi: 10.3389/fpls.2023.1290600 (PMC10690955; doi:10.3389/fpls.2023.1290600)
Supplement: Supplementary file 1 [file Image_1.pdf]

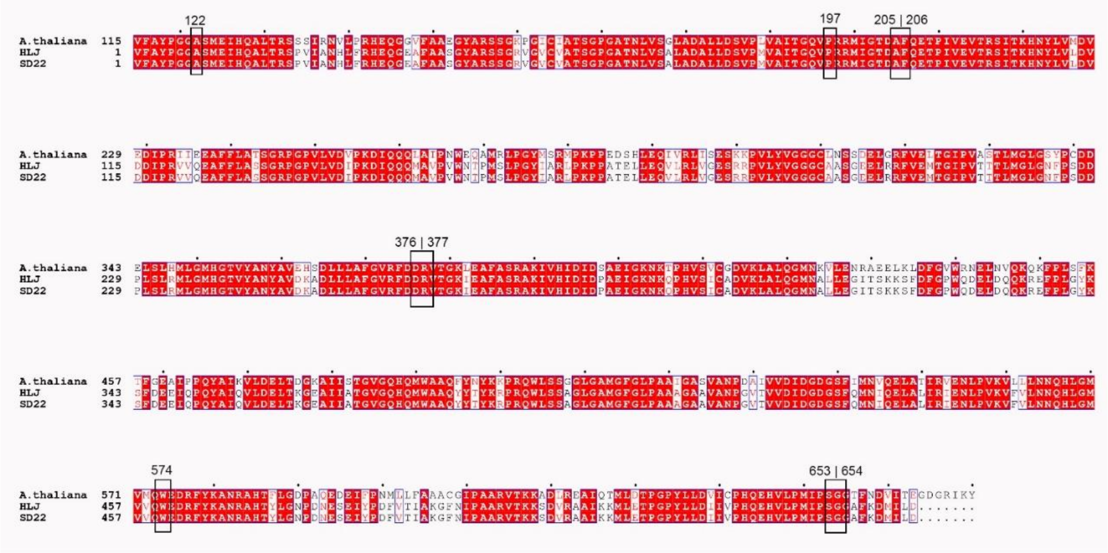

Supplementary Figure 1. Alignment of predicted amino acid sequences of *Digitaria sanguinalis* acetolactate synthase. The black boxes indicate the nine amino acid substitution sites known to be associated with weed resistance (numbered according to corresponding sequence of *Arabidopsis thaliana*).
