# Supplementary material for: ALS gene overexpression and enhanced metabolism conferring Digitaria sanguinalis resistance to nicosulfuron in China
Source: Front Plant Sci. 2023 Nov 17;14:1290600. doi: 10.3389/fpls.2023.1290600 (PMC10690955; doi:10.3389/fpls.2023.1290600)
Supplement: Supplementary file 2 [file Table_1.docx]

**Supplementary Table 1.**  Survival rate of different *Digitaria sanguinalis* populations after nicosulfuron treatment

| Population | Dose^a^ (g a.i. ha^−1^) | Total number of plants | number of death | Survival rate (%) ±SE |
| --- | --- | --- | --- | --- |
| HLJ | 30 | 24 | 0 | 100.00±0.00 |
|  | **60** | 24 | 0 | 100.00±0.00 |
|  | 120 | 24 | 3 | 87.50±0.07 |
| BJ | 30 | 24 | 0 | 100.00±0.00 |
|  | **60** | 24 | 2 | 91.67±0.08 |
|  | 120 | 24 | 3 | 87.50±0.13 |
| JL | 30 | 24 | 0 | 100.00±0.00 |
|  | **60** | 24 | 6 | 75.00±0.07 |
|  | 120 | 24 | 15 | 37.50±0.13 |
| AH | 30 | 24 | 1 | 95.83±0.04 |
|  | **60** | 24 | 3 | 87.50±0.13 |
|  | 120 | 24 | 10 | 58.33±0.11 |
| SD22 | 30 | 24 | 7 | 70.83±0.04 |
|  | **60** | 24 | 12 | 50.00±0.04 |
|  | 120 | 24 | 100 | 0.00±0.00 |

^a^ Bold numbers indicate the recommended field dose
