# Supplementary material for: ALS gene overexpression and enhanced metabolism conferring Digitaria sanguinalis resistance to nicosulfuron in China
Source: Front Plant Sci. 2023 Nov 17;14:1290600. doi: 10.3389/fpls.2023.1290600 (PMC10690955; doi:10.3389/fpls.2023.1290600)
Supplement: Supplementary file 3 [file Table_2.docx]

**Supplementary Table 2.** Parameters used to evaluate the reference genes.

| Parameters | Reference gene | | | |
| --- | --- | --- | --- | --- |
|  | *UBQ* | *Actin* | *GAPDH* | *18S rRNA* |
| Sample number | 16 | 16 | 16 | 16 |
| Geometrical mean of CT value | 22.71 | 28.17 | 28.07 | 16.61 |
| Arithmetic mean of CT value | 22.73 | 28.20 | 28.18 | 16.74 |
| Minimum | 21.22 | 25.85 | 24.70 | 13.45 |
| Minimum | 24.38 | 31.13 | 33.11 | 20.37 |
| Standard deviation | 0.72 | 1.00 | 2.20 | 1.78 |
| Coefficient of variation (%) | 3.15 | 3.54 | 7.80 | 10.62 |
